# Effect of Ethyl Acetate Fraction from *Eucommia ulmoides* Leaves on PM_2.5_-Induced Inflammation and Cognitive Dysfunction

**DOI:** 10.1155/2022/7157444

**Published:** 2022-05-14

**Authors:** Min Ji Kim, Jin Yong Kang, Jong Min Kim, Jong Hyun Moon, Hyo Lim Lee, Hye Rin Jeong, Min Ji Go, Uk Lee, Ho Jin Heo

**Affiliations:** ^1^Division of Applied Life Science (BK21), Institute of Agriculture and Life Science, Gyeongsang National University, Jinju 52828, Republic of Korea; ^2^World Institute of Kimchi an Annex of Korea Food Research Institute, Gwangju, Republic of Korea; ^3^Division of Special Forest Products, National Institute of Forest Science, Suwon 16631, Republic of Korea

## Abstract

This study aimed to evaluate the protective effect of the ethyl acetate from *Eucommia ulmoides* leaves (EFEL) on PM_2.5_-induced cognitive impairment in BALB/c mice. EFEL improved PM_2.5_-induced cognitive decline by improving spontaneous alternative behavioral and long-term memory ability. EFEL increased ferric reducing activity power (FRAP) in serum. In addition, EFEL increased superoxide dismutase (SOD) and reduced glutathione (GSH) contents and inhibited the production of malondialdehyde (MDA) in lung and brain tissues. EFEL also restored the mitochondrial function by regulating reactive oxygen species (ROS) production, mitochondrial membrane potential (MMP) level, and ATP level in lung and brain tissues. EFEL ameliorated the cholinergic system by regulating the acetylcholine (ACh) content and acetylcholinesterase (AChE) activity in the brain tissue and the expression of AChE and choline acetyltransferase (ChAT) in the whole brain and hippocampal tissues. EFEL reduced PM_2.5_-induced excessive expression of inflammatory protein related to the lung, whole brain, olfactory bulb, and hippocampus. Physiological compounds of EFEL were identified as 5-O-caffeolyquinic acid, rutin, quercetin, and quercetin glycosides. As a result, EFEL has anti-inflammation and anti-amnesic effect on PM_2.5_-induced cognitive impairment by regulating the inflammation and inhibiting the lung and brain tissue dysfunction, and its effect is considered to be due to the physiological compounds of EFEL.

## 1. Introduction

Air pollution is an environmental issue that is becoming a global problem, and its harmful effect on the human body is gradually increasing [1]. According to the World Health Organization (WHO), air pollution causes stroke, heart disease, lung cancer, and respiratory diseases, resulting in approximately 3.0 million deaths annually [2]. Particulate matter (PM), one of the causes of air pollution, is generated from construction sites, factories, and automobile exhaust gas and is composed of organic ions such as sulfate, nitrate, and ammonium ion, carbon, and mineral components [3]. PM is classified as PM_10_, which is smaller than 10 *μ*m in diameter, and PM_2.5_, which is smaller than 2.5 *μ*m in diameter [4]. PM_2.5_ can easily reach the bronchi and lungs because it cannot be filtered by the cilia. The PM_2.5_ deposited in the respiratory system causes reactive oxygen species (ROS), oxidative stress, inflammation, and apoptosis, inducing respiratory diseases such as asthma, pneumonia, chronic cough, disruption of calcium homeostasis, and mediating of inflammatory responses [5]. PM_2.5_ exposure increases the ROS and inflammation factors by oxidative stress and inflammatory response in lung tissue [6]. Also, PM_2.5_ that has penetrated into the alveoli can pass through the air-blood barrier and circulate throughout the body, where it can be deposited in the brain tissue [7]. Also, inflammatory cytokines produced by the inflammation of lung tissue circulate whole body through blood and affect the central neuronal system [8]. As a result, inflammation induced by nanoparticles exposure leads to neuronal dysfunction such as loss of neurons and disruption of synapses, resulting in learning and memory impairment [9]. In addition, PM_2.5_ directly reaches the olfactory bulb, passing through the blood-brain barrier (BBB), which induces nervous system dysfunction and causes neuroinflammation and neurodegenerative diseases [10]. The PM_2.5_ absorbed induces an excessive inflammatory response, which leads to cell apoptosis in the brain by producing ROS and inflammatory cytokines [11].

*Eucommia ulmoides* is a plant in the family *Eucommiaceae* and is widely cultivated in China, Japan, and Korea [12]. *Eucommia ulmoides* has various compounds such as flavonoids, lignans, iridoids, and phenols, and its barks and leaves are used as herbal medicines for the prevention of hypertension, diabetes, and hepotoxicity [13–15]. According to previous studies, *Eucommia ulmoides* leaves extract decreased the levels of total cholesterol, LDH cholesterol, and free fatty acids in serum and decreased the activity of hepatic fatty acid synthase and HMG-CoA enzyme in a high-fat diet-induced hamster model [16]. The water extract of *Eucommia ulmoides* leaves showed antioxidant activity in the liver of a CCl_4_-induced hepatotoxicity model and decreased GOT, GPT, LDH, and ALP levels in serum [15]. *Eucommia ulmoides* extract improved motor dysfunction in 1-methyl-4-phenyl-1,2,3,6-tetrahydropyridine (MPTP-)-induced Parkinson's model mice through pole and rotarod tests and increased the levels of dopamine and its metabolites such as 3,4-dihydroxyphenylacetic acid and homovanillic acid in striatum. Also, *Eucommia ulmoides* extract showed a protective effect on neuroinflammation by reducing the levels of TNF-*α*, IL-1*β*, and IL-6 in the blood [17]. In addition, it was confirmed that *Eucommia ulmoides* improved the neurotoxicity of amyloid beta-induced-PC12 cells [18]. However, the studies on the physiological activities of the *Eucommia ulmoides* leaves are mainly focused on the improvement effect of hepatoxicity and lipid metabolism, especially there are few studies on *Eucommia ulmoides* leaves related to the effect on PM_2.5_-induced inflammation and cognitive dysfunction. Therefore, in this study, anti-inflammatory and amnesic effects were confirmed using the ethyl acetate fraction from *Eucommia ulmoides* leaves in PM_2.5_-induced BALB/c mice, and the potential availability as a functional food material for the prevention of PM_2.5_-induced cognitive impairment was evaluated.

## 2. Materials and Methods

### 2.1. Sample Preparation

*Eucommia ulmoides* leaves were purchased from Yeongcheon, Gyeongsangbuk-do, Korea, in April 2019 and verified by the National Institute of Forest Science (Suwon, Korea). The sample was extracted in 40% ethanol for 2 h at 40°C and evaporated using a vacuum rotary evaporator (N-N series, Eyela Co., Tokyo, Japan). After that, the extract was sequentially fractionated using *n*-hexane, chloroform, and ethyl acetate and lyophilized. According to previous study, ethyl acetate fraction from *Eucommia ulmoides* leaves (EFEL) was presented as the significant total phenolic and flavonoid contents and antioxidant activities, and it was used for this study (Supplementary Figures 1 and 2) [19].

### 2.2. Animal Experiment Design

The experimental animals (BALB/c, 6-week, male) were bred in certain temperature (22 ± 2°C) and humidity (50 ± 5%) conditions. All experiments were performed according to the guidelines of Animal Care and Use Committee of the Gyeongsang National University (approval number: GNU-200302-M0007; approval day: 03/02/2020). Mice were randomly divided to 4 groups: control (clean air exposure), PM_2.5_ (PM_2.5_ exposure), EFEL 20 (PM_2.5_ exposure + EFEL 20 mg/kg of body weight), and EFEL 40 (PM_2.5_ exposure + EFEL 40 mg/kg of body weight) (*n* = 20 per group; 7 for *in vivo* tests; 5 for *ex vivo* tests; 5 for mitochondrial activity tests; and 3 for western blot analysis). PM_2.5_, EFEL 20, and EFEL 40 groups were exposed to PM_2.5_ at a constant concentration (500 *μ*g/m^3^) in whole body exposure chamber, and the control group was exposed to filtered air the same time. Clean air and PM_2.5_ were exposed 5 hours per day for 12 weeks. EFEL was dissolved in clean drinking water according to the dose and ingested in mice by oral administration once a day for 12 weeks.

### 2.3. In Vivo Behavioral Tests

#### 2.3.1. Y-Maze Test

The Y-maze consists of the length (33 cm), height (15 cm), and width (10 cm), respectively. Mice were placed at the end of one arm, and the movement of each mouse was recorded by a smart video tracking system (Smart 3.0, Panlab, Barcelona, Spain) for 8 min [20, 21]. In Y-maze test, evaluated behavioral parameters were total distance and triplet (%) of spontaneous alternative behavior. Total distance was similar in all groups, indicating that all mice were behaviorally normal. Triplet measures the sequential entry into the three arms of the maze.

#### 2.3.2. Morris Water Maze Test

Morris water maze consists of stainless circular pool (90 cm in diameter and 30 cm deep) and divided into quadrants (N, S, E, and W zones) with visual clues, and a platform was placed in the center of the W zone. In Morris water maze test, evaluated behavioral parameters were escape times for platform in W zone of pool through hidden tests and retention time in W zone through probe test. In the hidden test, the escape time to the platform was measured for 4 days (maximum time: 60 sec). In the probe test, the platform was removed retention time in the W zone, and movements of mice were recorded using a smart video tracking system (Smart 3.0, Panlab) [21, 22]. After all in vivo tests were completed, mice were sacrificed using CO_2_ inhalation.

### 2.4. Serum FRAP Analysis

After the behavioral test, the serum obtained by centrifugation at 10,000×*g* for 10 min at 4°C was used to measure ferric reducing/antioxidant power (FRAP). Serum was reacted with FRAP reagent consisting of 10 mM 2-4-5-tripyridyl-S-triazine (TPTZ): 20 mM FeCl_3_: 0.3 M sodium acetate buffer (pH 3.6) (1 : 1 : 10), and then, the reaction was measured by a microplate reader at 593 nm.

### 2.5. Sample Preparation

The lung and brain homogenates using 10 volumes phosphate buffer saline (PBS, pH 7.4) were used for evaluation of antioxidant system such as superoxide dismutase (SOD) and malondialdehyde (MDA) contents, and cholinergic system, such as acetylcholine (ACh) and acetylcholinesterase (AChE), and homogenate using 10 mM phosphate buffer with 1 mM EDTA (pH 6.0) were used for evaluation of reduced glutathione (GSH) content.

### 2.6. Antioxidant System Analysis

#### 2.6.1. SOD Contents

The supernatant obtained by centrifuging the lung and brain homogenates at 400×*g* for 10 min at 4°C was extracted using 10 volumes of cell extraction buffer. The extracted samples were centrifuged at 14,000×*g* for 5 min at 4°C, and the supernatants were used for the experiment according to manufacturer's protocol (Dojindo Molecular Technologies, Rockville, MD, USA). The absorbance was measured at 450 nm (Epoch2, BioTek, Winooski, VT, USA) [21, 23].

#### 2.6.2. Reduced GSH Contents

The supernatants obtained by centrifuging at 10,000×*g* for 15 min at 4°C of lung and brain homogenate were mixed with 5% metaphosphoric acid (1 : 1), and the mixtures were centrifuged at 2,000×*g* for 2 min at 4°C. The supernatants were mixed with 0.26 M tris-HCl, 0.65 N NaOH, and 1 M o-phthaldialdehyde and reacted for 15 min. Fluorescence was measured by a fluorescence microplate reader (Infinite 200, Tecan Co., San Jose, CA, USA) at emission (420 nm) and excitation (320 nm) wave [21, 24].

#### 2.6.3. MDA Production

The supernatants obtained by centrifuging at 5,000 rpm for 10 min at 4°C of lung and brain homogenate were reacted with 1% phosphoric acid and 0.67% TBA in water bath for 95°C for 1 h. The mixtures were spun down, and the absorbance of supernatant was measured at 532 nm (Epoch2, BioTek) [21, 25].

### 2.7. Cholinergic System

The supernatant obtained by centrifuging the brain tissue homogenate at 14,000×*g* for 30 min at 4°C was used for ACh levels and AChE activity [21, 26]. For ACh levels, 2 M hydroxylamine: 3.5 N NaOH (1 : 1) mixture was reacted with the obtained supernatant at room temperature, and then, 0.5 N HCl and 0.37 M FeCl_3_ were added, and the absorbance was measured at 540 nm using microplate reader (Epoch2, BioTek).

To evaluate the AChE activity, 50 mM sodium phosphate buffer was reacted with the obtained supernatant at 37°C for 15 min. After that, AChE solution composed to acetyl thiocholine and 5,5'dithio-bis (2-nitroenzoic acid) (DTNB) was added, and absorbance was measured at 405 nm using microplate reader (Epoch, BioTek).

### 2.8. Mitochondrial Extraction

Lung and brain tissues were homogenized into a mitochondrial isolation (MI) buffer including 215 mM mannitol, 75 mM sucrose, 0.1% bovine serum albumin (BSA), 20 mM HEPES (Na^+^), and 20 mM EDTA using a bullet blender (BBY24M, Next Advance Inc., Averill Park, NY, USA). The pellet obtained by centrifuge (13,000×*g*, 10 min at 4°C) was mixed with MI buffer containing 0.1% digitonin. The mitochondrial sample and MI buffer without 20 mM EDTA were mixed and centrifuged at 13,000×*g* for 15 min at 4°C. The pellet obtained was mixed with the EDTA-free MI buffer and centrifuged at 10,000×*g* for 10 min at 4°C [21, 27]. The obtained pellet mixed with EDTA-free MI buffer was used to measure ROS content and mitochondrial membrane potential (MMP).

#### 2.8.1. Mitochondrial ROS Production

ROS content and MMP activity used quantified protein by Bradford assay [28]. The mitochondrial extract (0.80 mg/mL) was used to measure ROS contents, and the mitochondrial extract and 25 *μ*M dichlorofluorescin diacetate (DCF-DA) reagent were mixed and reacted in the dark for 20 min. Fluorescence of mixture was measured by a fluorescence microplate reader (Infinite 200, Tecan Co.) at emission (485 nm) and excitation (535 nm) wave.

#### 2.8.2. Mitochondrial Membrane Potential

The mitochondrial extract (1.20 mg/mL) was used to measure MMP activity, and the mitochondrial extract was reacted with EDTA-free MI buffer containing 5 mM pyruvate and malate and 1 *μ*M tetraethylbenzimidazolyl-carbocyanine iodide (JC-1) in the dark for 20 min. Fluorescence of mixture was measured by a fluorescence microplate reader (Infinite 200, Tecan Co.) at emission (535 nm) and excitation (590 nm) wave.

#### 2.8.3. Mitochondrial ATP Content

The mitochondrial extract was quantified at the same concentration by Bradford assay. The pellet obtained by centrifugation (10,000×*g* for 10 min at 4°C) was reacted with 1% trichloroacetic acid (TCA), and it was mixed with 25 mM Tris-acetate buffer (pH 7.7) and centrifuged at 10,000×*g* for 15 min at 4°C. The obtained supernatants were measured for ATP level using ATP assay kit (Promega, Corp., Madison, WI, USA).

### 2.9. Western Blot Assay for Protein Expression

After mice brain tissue removal, olfactory bulb and hippocampal tissue were isolated. Lung, whole brain, olfactory bulb, and hippocampus tissues were homogenized with ProtinExTM animal cell/tissue (Gene All Biotechnology, Seoul, Korea) containing 1% protease inhibitor cocktails (Thermo Fisher Scientific, Rockford, IL, USA). And homogenates were immediately centrifuged at 13,000×*g* for 10 min at 4°C. The obtained supernatants were quantified with the same concentration protein using a Bradford reagent (Bio-rad, Hercules, CA, USA) according to Bradford assay. After that, supernatants were mixed with 1× loading dye and boiled at 95°C for 5 min in water bath. The proteins were separated on sodium dodecyl sulfate polyacrylamide gel (SDS-PAGE) and transferred to a polyvinylidene difluoride (PVDF) membrane (Millipore, Billerica, MA, USA). The membranes were blocked with 5% skim milk solution and incubated with primary antibody (1 : 1000) for overnight at 4°C. After incubation, the membranes were incubated with secondary antibody (1 : 2000) for 1 h at room temperature [21]. Finally, protein expression was detected with ProNA™ ECL Ottimo (TransLab, Daejeon, Korea) using an iBright CL 1000Imaging System (Thermo Fischer Scientific, Rockford, IL, USA). The density of the expressed protein was quantified using the iBright Analysis System (Thermo Fischer Scientific).

### 2.10. Identification of Physiologically Compounds Using UPLC Q-TOF/MS^2^

To identify the physiologically compounds of EFEL, ultraperformance liquid chromatography-ion mobility separation-quadrupole time of flight/tandem mass spectrometry (UPLC-Q-TOF/MS^2^, Vion, Waters Corp., Milford, MA, USA) was performed. EFEL was dissolved in 100% methanol and filtered using 0.2 *μ*m membrane filters. UPLC separation was performed using an ACQUITY UPLC BEH C_18_ column (2.1 × 100 mm and 1.7 *μ*m particle size, Waters Corp.). The flow rate was 0.60 mL/min. The mobile phases were composed as solvent A (0.1% formic acid in distilled water) and solvent B (0.1% formic acid in acetonitrile), and the analysis conditions were conducted as follows: a gradient elution of 40% A and 60% B at 0–1 min, 40% A and 60% B at 1–8 min, 100% B at 8–9 min, 100% B at 9-9.50 min, and 40% A and 60% B at 9.50–12 min. The conditions of negative electrospray ionization (ESI) were conducted as follows: ramp collision energy, 10–30 V; capillary voltage, 2.5 kV; source temperature, 100°C; desolvation temperature, 250°C; and mass range, 50–1000 m/z.

### 2.11. Statistical Analysis

All experiment results were expressed as mean ± standard deviation (SD). The mean value was verified for significance differences by Duncan's multiple range test (*p* < 0.05) using SAS software (Version 9.4, SAS institute, Cary, NC, USA).

## 3. Results

### 3.1. *In Vivo* Behavior *Tests*

#### 3.1.1. Y-Maze Test

**Figure 1:**
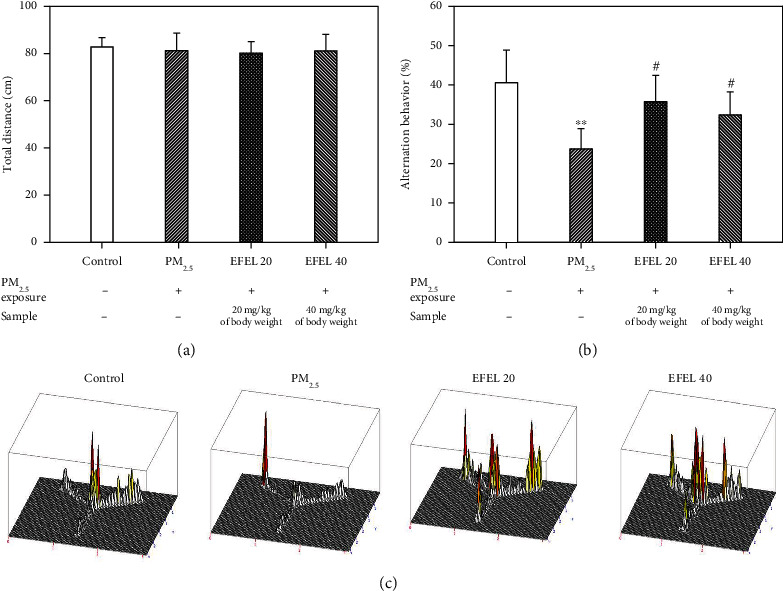
Protective effect of ethyl acetate fraction from *Eucommia ulmoides* leaves (EFEL) in PM_2.5_-induced mice. (a) Number of arm entries; (b) spontaneous alternation behavior; and (c) 3D moving path in Y-maze test. Results shown are mean ± SD (*n* = 7). Data were statistically represented at ∗ which is significantly different from the control group and # which is significantly different from PM_2.5_ group; ∗ and #*p* < 0.05; and ∗∗and ##*p* < 0.01.

In the Y-maze test, the total distance of each mice showed no significant difference in all groups (Figure 1(a)) (control: 85.25 ± 3.28; PM_2.5_ group: 79.88 ± 7.87; EFEL 20 group: 79.14 ± 6.45; and EFEL 40 group: 80.66 ± 4.83). These results indicated that all mice had the same motor performance. In the results of spontaneous alternation behavior, the PM_2.5_ group (23.71% ± 5.13 (*p* value 0.00)) showed reduced spontaneous alternation behavior compared to the control group (40.54% ± 8.25). However, the EFEL groups showed increased spontaneous alternation behavior compared to the PM_2.5_ group (EFEL 20 group: 33.56% ± 6.67 (*p* value 0.02); EFEL 40 group: 32.33% ± 5.82 (*p* value 0.06)) (Figure 1(b)).

#### 3.1.2. Morris Water Maze Test

**Figure 2:**
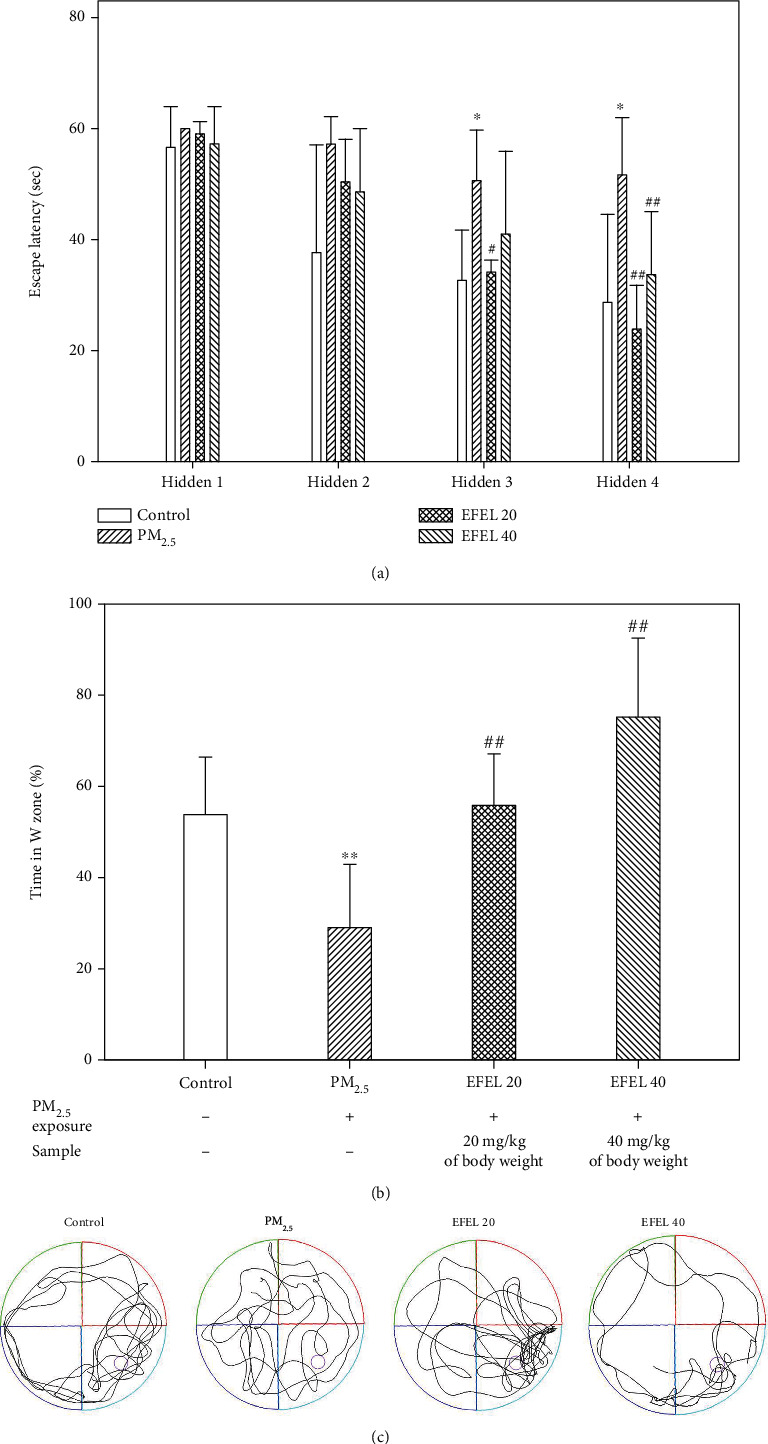
Protective effect of ethyl acetate fraction from *Eucommia ulmoides* leaves (EFEL) in PM_2.5_-induced mice. (a) Escape latency; (b) time in W zone; and (c) 2D moving path in Morris water maze test. Results shown are mean ± SD (*n* = 7). Data were statistically represented at ∗ which is significantly different from the control group and #which is significantly different from PM_2.5_ group; ∗ and #*p* < 0.05; and ∗∗ and ##*p* < 0.01.

The results of the Morris water maze test are shown in Figure 2. It was confirmed that the escape time of the PM_2.5_ group (51.70 sec ± 10.31 (*p* value 0.04)) was longer than the control group (28.75 sec ± 15.87). The escape times of the EFEL groups (EFEL 20 group, 23.94 sec ± 7.91 (*p* value 0.01); EFEL 40 group, 33.78 sec ± 11.31 (*p* value 0.01)) were shorter compared to the PM_2.5_ group. In the probe test, the retention time in the W zone of the PM_2.5_ group (29.11% ± 13.85 (*p* value 0.00)) was decreased compared to the control group (53.80% ± 12.64). However, the EFEL groups (EFEL 20 group, 55.85% ± 11.26 (*p* value 0.01); EFEL 40 group, 75.16% ± 17.28 (*p* value 0.00)) increased the retention time in the W zone compared to the PM_2.5_ group (Figure 2(b)).

### 3.2. Serum FRAP Analysis

**Figure 3:**
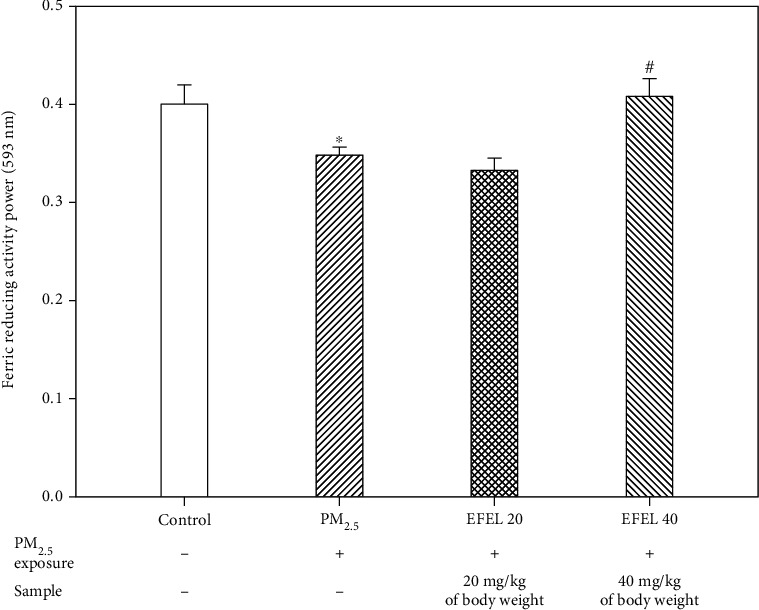
Ferric reducing/antioxidant power of ethyl acetate fraction from *Eucommia ulmoides* leaves (EFEL) on PM_2.5_-induced mice in serum. Results shown are mean ± SD (*n* = 3). Data were statistically represented at ∗which is significantly different from the control group and #which is significantly different from PM_2.5_ group; ∗ and #*p* < 0.05; and ∗∗and ##*p* < 0.01.

Serum FRAP activity is shown in Figure 3. Serum FRAP activity was lowered in the PM_2.5_ group (0.35 ± 0.008 (*p* value 0.02)) and improved in the EFEL groups (EFEL 20, 0.33 ± 0.01 (*p* value 0.12); and EFEL 40, 0.41 ± 0.02 (*p* value 0.02)) similarly to the control group (0.40 ± 0.02).

### 3.3. Antioxidant System

**Figure 4:**
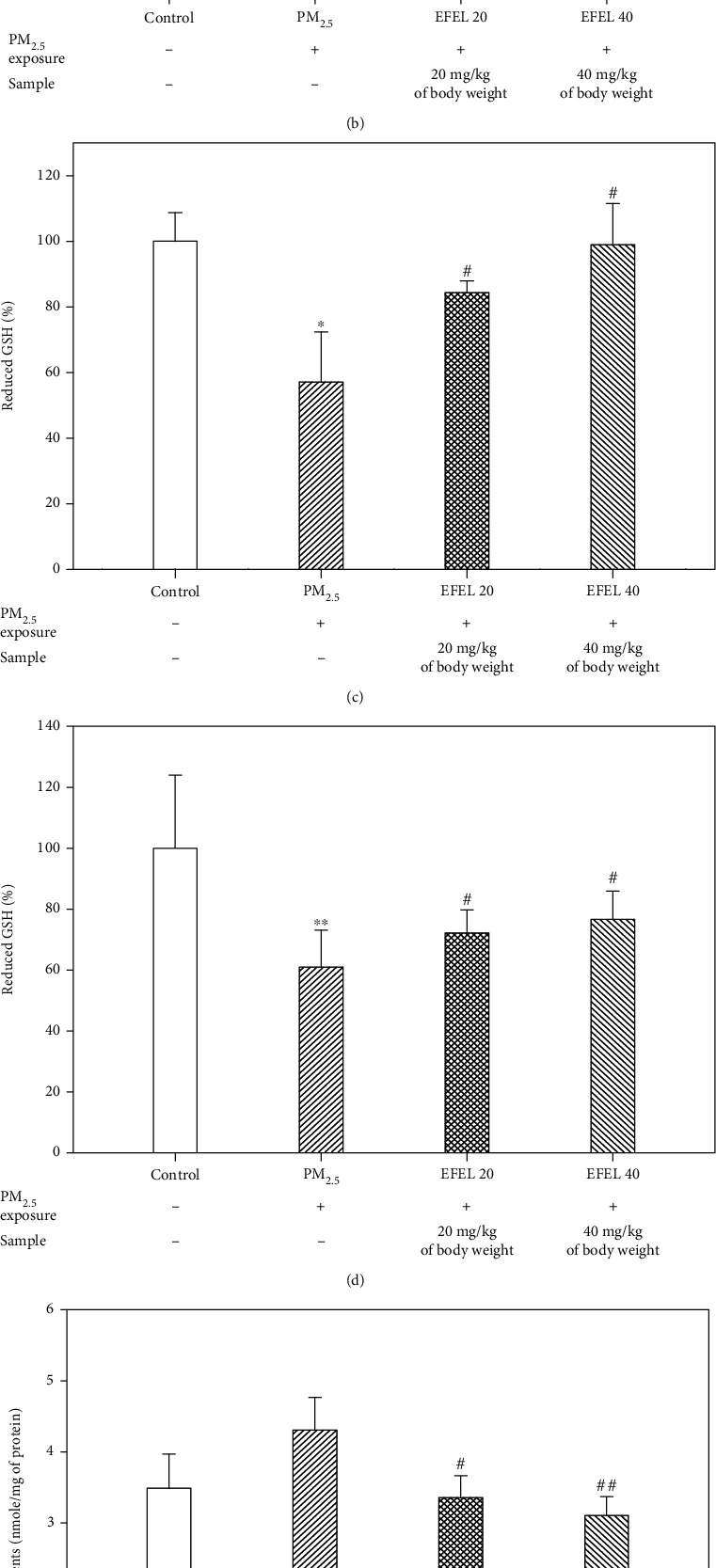
Protective effect of ethyl acetate fraction from *Eucommia ulmoides* leaves (EFEL) on antioxidant system in PM_2.5_-induced mice. Superoxide dismutase (SOD) content in the brain (a) and lung (b). Reduced GSH content in the brain (c) and lung (d). Malondialdehyde (MDA) content in the brain (e) and lung (f). Results shown are mean ± SD (*n* = 5). Data were statistically represented at ∗which is significantly different from the control group and # which is significantly different from PM_2.5_ group; ∗ and #*p* < 0.05; and ∗∗ and ##*p* < 0.01.

The results of SOD, reduced GSH, and MDA content in the lung and brain tissues are presented in Figure 4. The SOD content in the lung tissue of the PM_2.5_ group (3.90 ± 0.56 unit/mg of protein (*p* value 0.21)) decreased compared to the control group (4.45 ± 0.30 unit/mg of protein). However, SOD contents of the EFEL groups (EFEL 20, 4.58 ± 0.20 unit/mg of protein (*p* value 0.03); EFEL 40, 5.76 ± 0.49 unit/mg of protein (*p* value 0.00)) were increased compared to the PM_2.5_ group (Figure 4(a)). The reduced GSH content (61.00 ± 12.12% of control (*p* value 0.01)) in lung tissues of the PM_2.5_ group decreased compared with the control group (100 ± 24.01%). However, the EFEL groups increased the reduced GSH content (EFEL 20, 72.19 ± 7.56% of control (*p* value 0.03); EFEL 40, 76.62 ± 9.28% of control (*p* value 0.03)) compared to the PM_2.5_ group (Figure 4(c)). MDA content in lung tissues of the PM_2.5_ group (1.47 ± 0.05 nmole/mg of protein (*p* value 0.02)) increased compared with the control group (1.34 ± 0.04 nmole/mg of protein). However, the EFEL groups had decreased MDA production (EFEL 20, 1.39 ± 0.07 nmole/mg of protein (*p* value 0.25); EFEL 40, 1.31 ± 0.08 nmole/mg of protein (*p* value 0.04)) compared to the PM_2.5_ group (Figure 4(e)).

The SOD content in the brain tissue of the PM_2.5_ group (3.50 ± 0.22 unit/mg of protein (*p* value 0.02)) decreased compared to the control group (4.10 ± 0.31 unit/mg of protein). It also decreased in the EFEL groups (EFEL 20, 4.53 ± 0.48 unit/mg of protein (*p* value 0.04); EFEL 40, 4.46 ± 0.36 unit/mg of protein (*p* value 0.04)) compared to the PM_2.5_ groups (Figure 4(b)). The reduced GSH content in the brain tissue of the PM_2.5_ group (57.11 ± 15.22% of control (*p* value 0.02)) decreased compared to the control group (100 ± 14.25%). However, the EFEL groups increased the reduced GSH content (EFEL 20, 84.38 ± 3.51% of control (*p* value 0.03); EFEL 40, 98.93 ± 12.55% of control (*p* value 0.02)) compared to PM_2.5_ (Figure 4(d)). MDA content in the brain tissue of the PM_2.5_ group (4.32 ± 0.46 nmole/mg of protein (*p* value 0.13)) increased compared with the control group (3.50 ± 0.47 nmole/mg of protein). The EFEL groups saw decreased MDA production (EFEL 20, 3.37 ± 0.30 nmole/mg of protein (*p* value 0.04); EFEL 40, 3.11 ± 0.26 nmole/mg of protein (*p* value 0.00)) compared to the PM_2.5_ group (Figure 4(f)).

### 3.4. Cholinergic System

**Figure 5:**
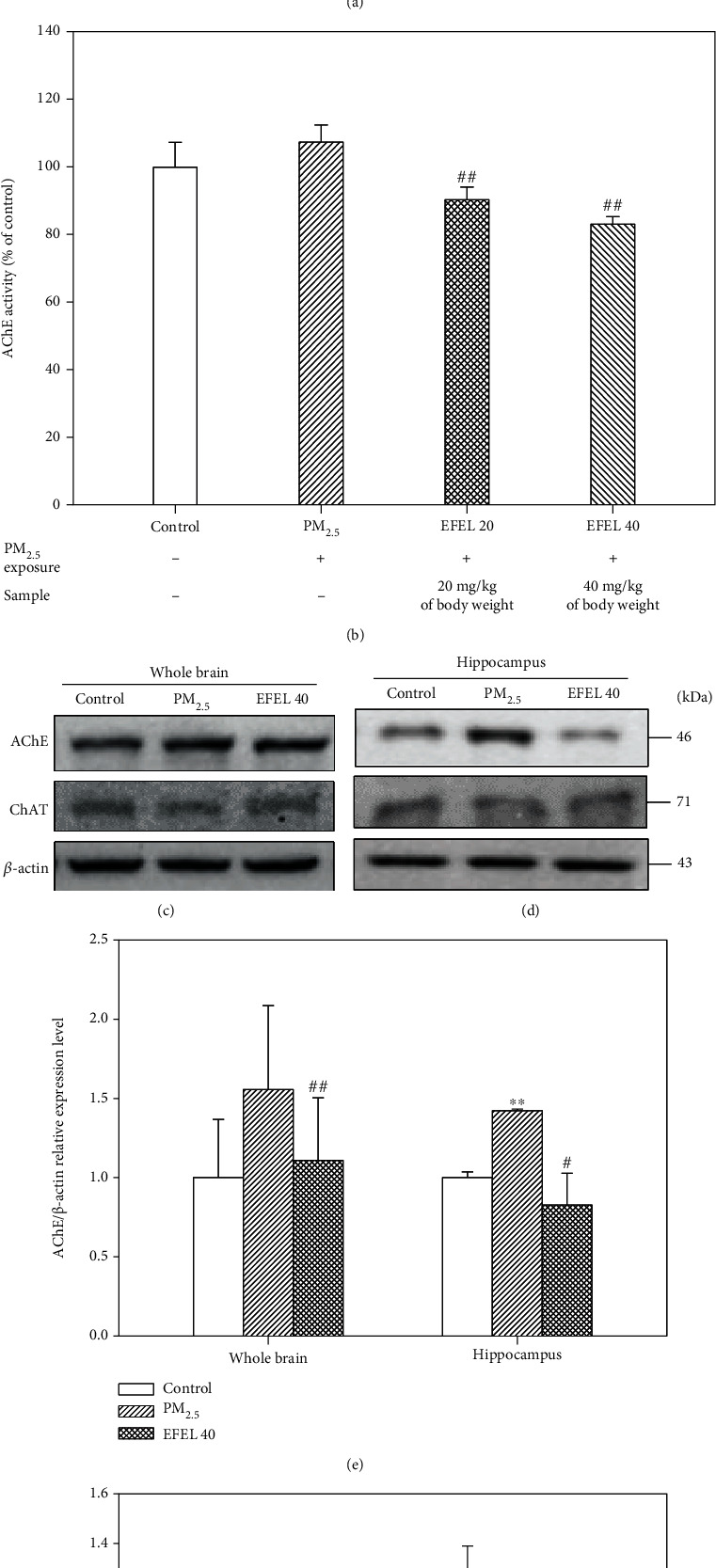
Protective effect of ethyl acetate fraction from *Eucommia ulmoides* leaves (EFEL) on cholinergic dysfunction in PM_2.5_-induced mice. (a) Acetylcholine (ACh) contents. (b) Acetylcholinesterase (AChE) activity in the whole brain. Acetylcholinesterase and choline acetyltransferase (ChAT) band image in the whole brain (c) and hippocampus (d). AChE and ChAT expression level in the whole brain (e and f) and hippocampal (g and h). Results shown are mean ± SD (*n* = 5). Data were statistically represented at ∗ which is significantly different from the control group and # which is significantly different from PM_2.5_ group; ∗ and #*p* < 0.05; and ∗∗ and ##*p* < 0.01.

The cholinergic system with acetylcholinesterase (AChE) and choline acetyltransferase (ChAT) is shown in Figure 5. PM_2.5_ significantly increased the activity of AChE (108.52 ± 4.07% of control (*p* value 0.14)), and the EFEL groups (EFEL 20, 91.37 ± 3.79% of control (*p* value 0.01); EFEL 40, 83.35 ± 1.47% of control (*p* value 0.00)) decreased the activity of AChE. In addition, PM_2.5_ decreased ACh content (1.68 ± 0.08 mM/mg of protein (*p* value 0.00)) compared with the control group (2.18 ± 0.17 mM/mg of protein). And the EFEL groups (EFEL 20, 1.71 ± 0.06 mM/mg of protein (*p* value 0.53); EFEL 40, 1.95 ± 0.07 mM/mg of protein (*p* value 0.02)) increased ACh content compared with the PM_2.5_ group (Figures 5(a) and 5(b)). In addition, Figures 5(c) and 5(d) show band images of the whole brain and hippocampal expression levels of AChE and ChAT acting on the degradation and synthesis of ACh, respectively. PM_2.5_ showed an increased AChE expression level (whole brain, 1.28 ± 0.14 (*p* value 0.08); hippocampus, 1.42 ± 0.01 (*p* value 0.00)) compared with the control group (whole brain, 1.00 ± 0.11; hippocampus, 1.00 ± 0.04). However, EFEL 40 decreased the AChE expression level (whole brain, 0.81 ± 0.07 (*p* value 0.01); hippocampus, 0.83 ± 0.20 (*p* value 0.04)). Also, PM_2.5_ showed a reduced ChAT expression level (whole brain, 0.33 ± 0.14 (*p* value 0.05); hippocampus, 0.58 ± 0.21 (*p* value 0.10)) compared with the control group (whole brain, 1.00 ± 0.27; hippocampus, 1.00 ± 0.39). However, EFEL 40 increased the ChAT expression level (whole brain, 1.01 ± 0.24 (*p* value 0.02); hippocampus, 0.77 ± 0.17 (*p* value 0.08)) (Figures 5(e) and 5(f)).

### 3.5. Mitochondrial Activity

**Figure 6:**
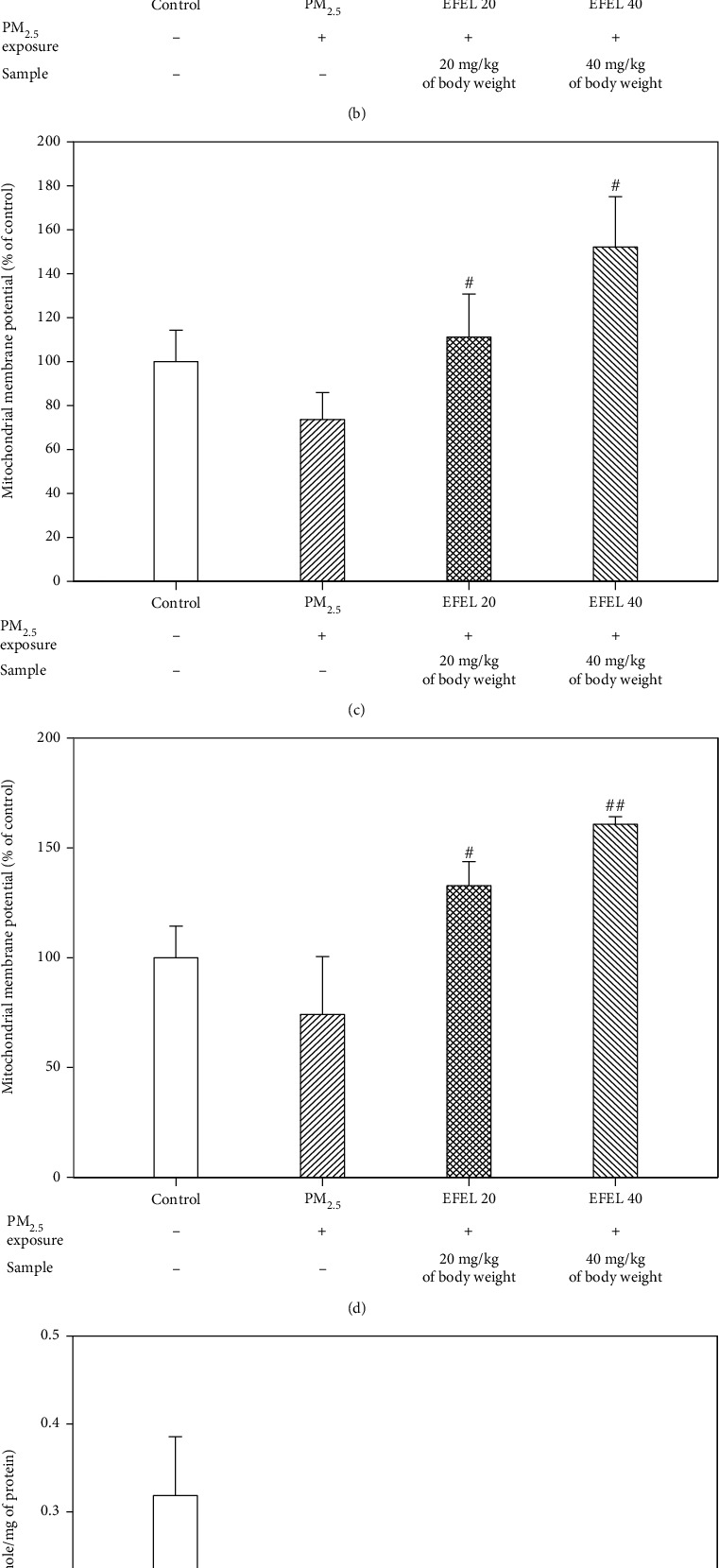
Mitochondrial activity of ethyl acetate fraction from *Eucommia ulmoides* leaves (EFEL) on PM_2.5_-induced mice in brain and lung. Mitochondrial reactive oxygen species (ROS) contents in the brain (a) and lung (b). Mitochondrial membrane potential (MMP) activity in the brain (c) and lung (d). Mitochondrial ATP contents in the brain (e) and lung (f). Results shown are mean ± SD (*n* = 5). Data were statistically represented at ∗ which is significantly different from the control group and # which is significantly different from PM_2.5_ group; ∗ and #*p* < 0.05; and ∗∗ and ##*p* < 0.01.

The ROS content, MMP and ATP level measured from the mitochondrial extract of mice lung and brain tissue are shown in Figure 6. The lungs of the PM_2.5_ group showed excessive ROS content (136.41 ± 25.46% of control (*p* value 0.04)), while EFEL 20 and 40 had decreased the ROS content (76.13 ± 15.57% of control (*p* value 0.05) and 61.11 ± 25.64% of control (*p* value 0.05)) (Figure 6(a)). MMP was decreased in the lungs of the PM_2.5_ group (74.13 ± 26.41% of control (*p* value 0.06)), and EFEL 20 and 40 had an increased MMP level (132.74 ± 11.03% of control (*p* value 0.04) and 160.73 ± 3.49% of control (*p* value 0.01)) (Figure 6(c)). The ATP level was decreased in the lungs of the PM_2.5_ group (0.17 ± 0.05 nmole/mg of protein (*p* value 0.04)) compared with the control group (0.31 ± 0.02 nmole/mg of protein), while the EFEL 20 and 40 groups showed 0.20 ± 0.05 nmole/mg of protein (*p* value 0.59) and 0.25 ± 0.03 nmole/mg of protein (*p* value 0.09), respectively (Figure 6(e)).

The brain of the PM_2.5_ group showed excessive ROS content (133.68 ± 16.92% of control (*p* value 0.05)), while EFEL 20 and 40 had decreased ROS content (80.62 ± 10.60% of control (*p* value 0.01) and 73.12 ± 11.64% of control (*p* value 0.02), respectively) (Figure 6(b)). MMP was decreased in the brain of the PM_2.5_ group (73.59 ± 12.21% of control (*p* value 0.05)) and EFEL 20 and 40 have an increased MMP level (111.11 ± 19.68% of control (*p* value 0.01) and 152.17 ± 22.89% of control (*p* value 0.01), respectively) (Figure 6(d)). The ATP level decreased in the brain of the PM_2.5_ group (0.09 ± 0.02 nmole/mg of protein (*p* value 0.04)) compared with the control group (0.32 ± 0.07 nmole/mg of protein), and the EFEL 20 and 40 groups showed 0.08 ± 0.01 nmole/mg of protein (*p* value 0.41) and 0.10 ± 0.05 nmole/mg of protein (*p* value 0.73), respectively (Figure 6(f)).

### 3.6. Western Blot Analysis

#### 3.6.1. Inflammatory Cytokine Expression Level in the Lung

**Figure 7:**
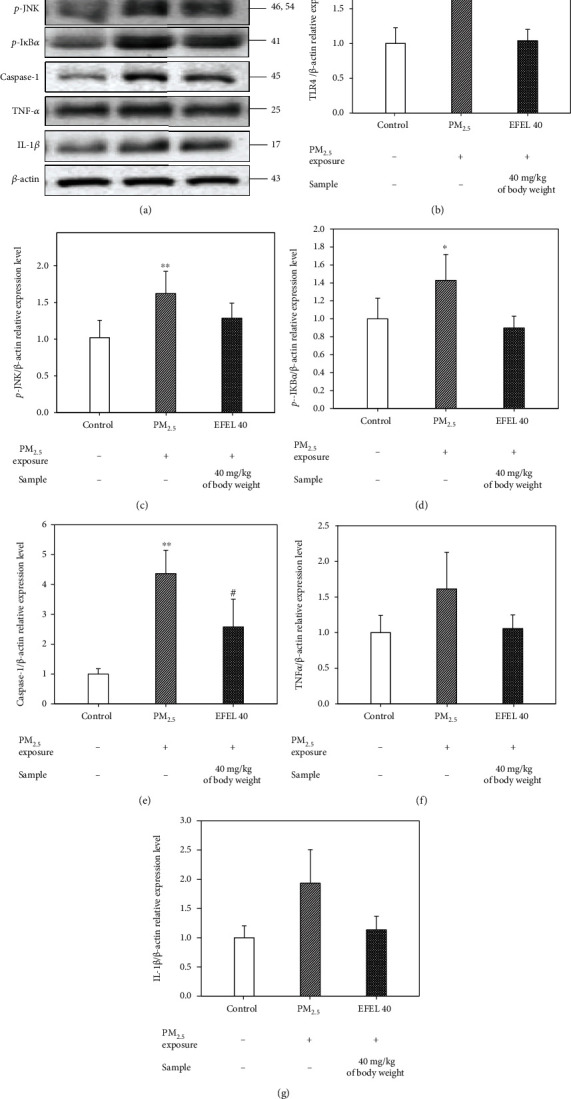
Expression levels of protein related to inflammation pathway in lung tissue. (a) band image; (b) TLR4; (c) *p*-JNK; (d) *p*-I*κ*b-*α*; (e) caspase-1; (f) TNF-*α*; and (g) IL-1*β*. Results shown are mean ± SD (*n* = 3). Data were statistically represented at ∗ which is significantly different from the control group and # which is significantly different from PM_2.5_ group; ∗ and #*p* < 0.05; and ∗∗ and ##*p* < 0.01.

Figure 7 shows the expression of protein mediating inflammatory responses in PM_2.5_-exposed mice lung tissue. The expression levels of TLR4, *p*-JNK, *p*-I*κ*B*α*, caspase-1, TNF-*α*, and IL-1*β* (63.64 ± 0.54% (*p* value 0.14), 58.75 ± 0.29% (*p* value 0.01), 42.84 ± 0.29% (*p* value 0.04), 336.12 ± 0.78% (*p* value 0.01), 61.37 ± 0.51% (*p* value 0.10), and 93.42 ± 0.57% (*p* value 0.09), respectively) in the PM_2.5_ group were up-regulated compared to the control group. However, the expression levels of the EFEL 40 group (59.65 ± 0.16% (*p* value 0.11), 45.81 ± 0.20% (*p* value 0.10), 53.04 ± 0.13% (*p* value 0.06), 178.49 ± 0.93% (*p* value 0.03), 55.78 ± 0.19% (*p* value 0.13), and 80.04 ± 0.23% (*p* value 0.12), respectively) were down-regulated compared to the PM_2.5_ group (Figures 7(b)–7(g)).

#### 3.6.2. Inflammation Protein Expression Level in the Whole Brain, Olfactory Bulb, and Hippocampus

**Figure 8:**
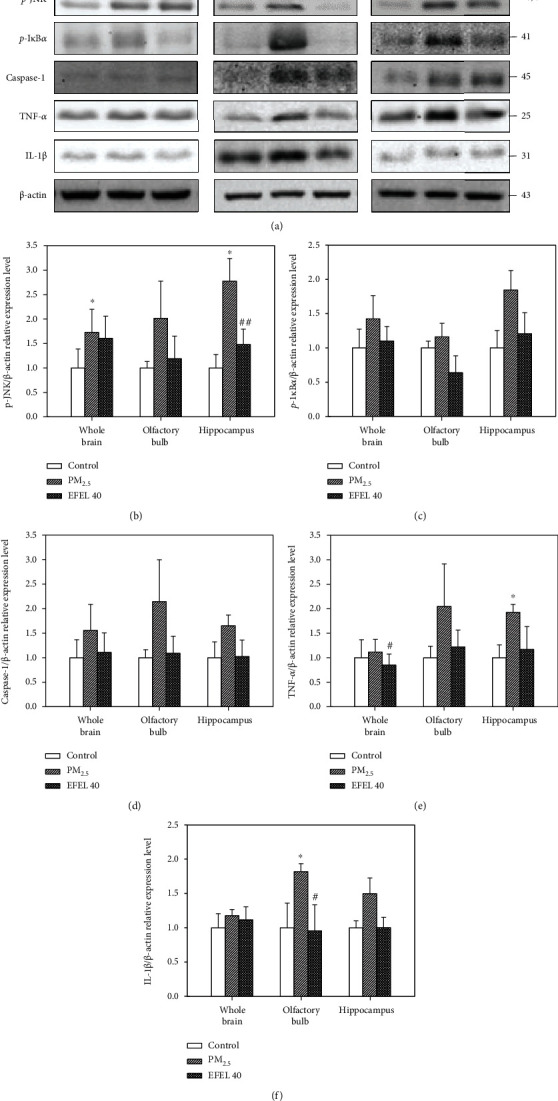
Expression levels of protein related to inflammation pathway in the whole brain, olfactory bulb and hippocampus tissues. (a) Band images; (b) *p*-JNK; (c) *p*-I*κ*B*α*; (d) caspase-1; (e) TNF-*α*; and (f) IL-1*β* relative expression level in olfactory bulb, whole brain, and hippocampus. Results shown are mean ± SD (*n* = 3). Data were statistically represented at ∗ which is significantly different from the control group and # which is significantly different from PM_2.5_ group; ∗ and #*p* < 0.05; and ∗∗ and ##*p* < 0.01.

Figure 8 shows the expression of protein-mediating inflammatory responses in PM_2.5_-exposed mice brain tissue. The expression levels of *p*-JNK (whole brain, 72.92 ± 0.47% (*p* value 0.02); olfactory bulb, 101.53 ± 0.76% (*p* value 0.10); and hippocampus, 177.29 ± 0.46% (*p* value 0.01)), *p*-I*κ*B*α* (whole brain, 42.36 ± 0.34% (*p* value 0.15); olfactory bulb, 16.16 ± 0.20% (*p* value 0.19); and hippocampus, 84.52 ± 0.28% (*p* value 0.07)), caspase-1 (whole brain, 55.55 ± 0.53% (*p* value 0.07); olfactory bulb, 114.27 ± 0.85% (*p* value 0.10); and hippocampus, 65.08 ± 0.22% (*p* value 0.15)), IL-1*β* (whole brain, 17.90 ± 0.09% (*p* value 0.15); olfactory bulb, 81.84 ± 0.12% (*p* value 0.04); and hippocampus, 49.81 ± 0.23% (*p* value 0.06)), and TNF-*α* (whole brain, 11.44 ± 0.26% (*p* value 0.55); olfactory bulb, 104.60 ± 0.87% (*p* value 0.12); hippocampus, 92.56 ± 0.16% (*p* value 0.04)) in the PM_2.5_ group were up-regulated compared to the control group. However, the expression levels of *p*-JNK (whole brain, 12.54 ± 0.46% (*p* value 0.49); olfactory bulb, 82.63 ± 0.47% (*p* value 0.07); and hippocampus, 129.49 ± 0.32% (*p* value 0.01)), *p*-I*κ*B*α* (whole brain, 32.28 ± 0.21% (*p* value 0.27); olfactory bulb, 52.20 ± 0.24% (*p* value 0.06); and hippocampus, 63.72 ± 0.31% (*p* value 0.16)), caspase-1 (whole brain, 44.71 ± 0.40% (*p* value 0.16); olfactory bulb, 105.39 ± 0.35% (*p* value 0.12); and hippocampus, 62.70 ± 0.33% (*p* value 0.18)), IL-1*β* (whole brain, 6.08 ± 0.19% (*p* value 0.59); olfactory bulb, 86.09 ± 0.38% (*p* value 0.04); and hippocampus, 49.54 ± 0.14% (*p* value 0.14)), and TNF-*α* (whole brain, 26.24 ± 0.22% (*p* value 0.01); olfactory bulb, 82.42 ± 0.34% (*p* value 0.09); and hippocampus, 75.44 ± 0.47% (*p* value 0.14)) in the EFEL 40 group were down-regulated compared to the PM_2.5_ group (Figures 8(b)–8(f)).

#### 3.6.3. Apoptosis Protein Expression Level in the Brain

**Figure 9:**
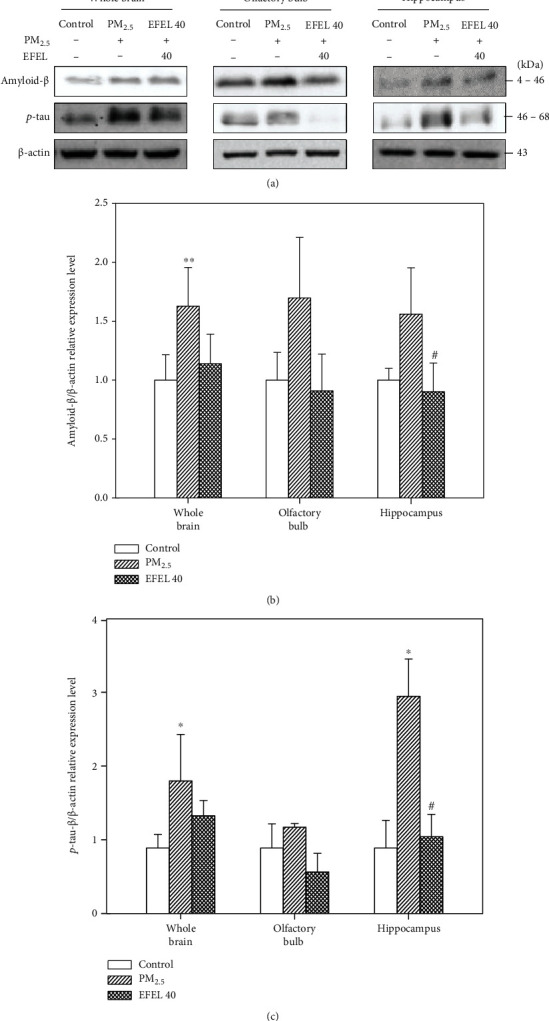
Expression levels of protein related to apoptosis in the whole brain, olfactory bulb, and hippocampus tissues. (a) Band images; (b) amyloid-*β*; and (c) *p*-tau relative expression in the whole brain, olfactory bulb, and hippocampus. Results shown are mean ± SD (*n* = 3). Data were statistically represented at ∗ which is significantly different from the control group and # which is significantly different from PM_2.5_ group; ∗ and #*p* < 0.05; and ∗∗ and ##*p* < 0.01.

Figure 9 shows the expression of amyloid-*β* and *p*-tau protein in mice whole brain, olfactory bulb, and hippocampus. The amyloid-*β* expression level increased 62.76 ± 0.33% (*p* value 0.00), 69.75 ± 0.51% (*p* value 0.08), and 56.00 ± 0.40% (*p* value 0.18) in the whole brain, olfactory bulb, and hippocampus compared to the control group, respectively. And EFEL decreased the expression level (whole brain, 48.89 ± 0.25% (*p* value 0.08); olfactory bulb, 78.83 ± 0.31% (*p* value 0.12); and hippocampus, 65.79 ± 0.24% (*p* value 0.02)) compared to the PM_2.5_ group. The *p*-tau expression level increased 101.62 ± 0.71% (*p* value 0.03), 31.90 ± 0.05% (*p* value 0.20), and 231.82 ± 0.57% (*p* value 0.02) in the whole brain, olfactory bulb, and hippocampus compared to the control group, respectively. And EFEL decreased the expression level (whole brain, 53.38 ± 0.21% (*p* value 0.22); olfactory bulb, 68.36 ± 0.28% (*p* value 0.03); and hippocampus, 214.58 ± 0.34% (*p* value 0.02)) compared to the PM_2.5_ group (Figures 9(b) and 9(c)).

### 3.7. Identification of Bioactive Compounds Using UPLC Q-TOF/MS^2^

**Figure 10:**
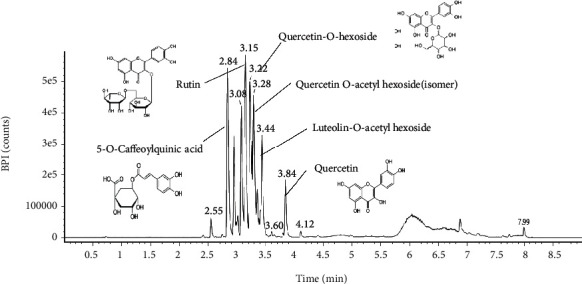
Ultraperformance liquid chromatography-ion mobility separation-quadrupole time of flight/tandem mass spectrometry (UPLC Q-TOF/MS^2^) chromatographic profile of ethyl acetate fraction from *Eucommia ulmoides* leaves (EFEL).

The major physiological compounds of EFEL were identified using UPLC IMS Q-TOF/MS^2^ analysis (Figure 10 and Table 1). The MS spectra were obtained in negative ion mode [M − H]^−^ as compound A, 707 m/z (RT: 2.84 min); compound B, 609 m/z (RT: 3.15 min); compound C, 463 m/z (RT: 3.22 min); compound D, 505 m/z (RT:3.28 min); compound E, 489 m/z (RT: 3.44 min); and compound F, 301 m/z (RT: 3.84 min). When the main fragments were compared with a previous study, these peaks were identified as 5-O-caffeoylquinic acid (compound A) [29], rutin (compound B) [30], quercetin-O-hexoside (compound C) [30], quercetin-O-acetylhexoside (isomer) (compound D) [31], luteolin-O-acetylhexoside (compound E) [29], and quercetin (compound F) [30].

## 4. Discussion

PM_2.5_ exposure induces oxidative stress and inflammation in respiratory system [32]. Because PM_2.5_ have small diameter, it is not filtered by the nasal mucosa and is deposited in the lungs and alveoli [5]. The release of proinflammatory cytokines (TNF-*α*, IL-6, and IFN-*γ*) produced by inflammation in the respiratory system, which circulate through the blood vessels throughout the body, results in systemic inflammation [32]. Also, PM_2.5_ and inflammatory cytokines that pass through the blood brain barrier (BBB) promote neuroinflammation, which leads to cognitive and learning deficits by neuronal damage, and PM_2.5_ can directly penetrate the olfactory bulb tissue through the olfactory nerve [33]. In this study, PM_2.5_ induced cognitive dysfunction, while EFEL improved the spontaneous alternative behavior and long-term memory ability of PM_2.5_-induced mice by regulating the inflammation and inhibited the tissues dysfunction in lung and brain tissues. *Eucommia ulmoides* bark showed neuroprotective effects by improving spontaneous alternative behavior and long-term memory ability in mice with amyloid-*β*-induced cognitive impairment [34]. Also, rutin as a bioactive substance within EFEL inhibited the long-term memory deficit in cadmium-induced rats, and quercetin improved the levels of spontaneous alternative behavior and short-term memory deficit in a TMT-induced mice model [35, 36]. In this study, EFEL containing quercetin and its derivatives improved spontaneous alternation behavior and long-term memory ability in PM_2.5_-induced cognitive dysfunction. Therefore, EFEL can be used as a material that improves cognitive dysfunction in PM_2.5_-induced mice.

PM_2.5_ can induce oxidative stress, which leads to a breakdown of the body's antioxidant system such as SOD and GSH. According to a study by Liu and Meng [37], the PM_2.5_ exposure decreased SOD and reduced GSH levels in the brain, lung, liver, and kidney of mice. Also, increased ROS due to an imbalance in the antioxidant system by PM_2.5_ exposure can also cause direct damage to cells and tissues [38]. The imbalance of the antioxidant system by ROS and PM_2.5_ can easily induce lipid peroxidation of unsaturated fatty acids in the brain tissue [39]. Similar to this study, PM_2.5_ reduced SOD and GSH levels and increased MDA levels in mice lung and brain tissues. However, EFEL inhibited the reduction of SOD and GSH levels and the production of MDA levels. The extract of *Eucommia ulmoides* bark increased the content of reduced GSH and the activity of catalase in the kidneys of cadmium-induced kidney toxicity mice [40] and increased the SOD and GSH-Px levels in the serum of streptozotocin-induced diabetic rats [41]. The macranthoin extracted from *Eucommia ulmoides* increased the level of SOD level and GSH content in H_2_O_2_-induced PC12 cells [42]. Also, the extract from *Eucommia ulmoides* leaves inhibited the production of MDA in the serum of streptozotocin-induced diabetic rats. Rutin increased the activities of SOD, GSH, catalase, and GPx on copper sulfate-induced brain damage of rats [43]. In addition, quercetin inhibited the MDA production on D-galactose-induced mice brain [44]. In this study, EFEL might not only regulate the reduction of SOD and GSH contents, but also inhibit the production of MDA. It was suggested that the intake of EFEL improved the PM_2.5_-induced damaged antioxidant system in lung and brain tissues.

The cholinergic system, which plays role the of neurotransmission in learning and memory ability, is highly correlated with oxidative stress and inflammation. Disruption of the cholinergic system due to increased inflammatory response leads to an abnormal neurotransmission system through the activation of the microglia and apoptosis pathway, and it is directly related to cognitive impairment [45]. PM_2.5_-induced oxidative stress and neuroinflammation induce disruption of the cholinergic system, which is a characteristic of neurodegenerative diseases such as Alzheimer's and Parkinson's diseases [46]. In this study, PM_2.5_-induced cholinergic system dysfunction and EFEL decreased the activity of AChE and increased the content of ACh and regulated the expression of AChE and ChAT in the whole brain and hippocampus tissues. The hippocampus functions related to learning and memory ability are easily affected by cholinergic dysfunction [47]. In the hippocampus tissues of cognitive impairment patients, acetylcholine (ACh) content was showed decreasing [48]. Therefore, the expression level of ChAT and AChE, which synthesize and degrade of ACh, were measured in the whole brain and hippocampus tissues. The bark of *Eucommia ulmoides* decreased AChE activity in the hippocampal and cortex of scopolamine-induced learning and cognitive impairment mice [49]. In addition, rutin as a physiological compound of *Eucommia ulmoides* leaves decreased AChE activity in the hippocampus and cortex of cadmium-induced rat [35]. Quercetin reduced the activity of AChE in the synaptosome, cerebral cortex, hippocampus, and striatum of the brain tissue on streptozotocin-induced diabetes rat model [50]. Also, quercetin regulated the mRNA expression levels of AChE and ChAT in the frontal cortex and hippocampus tissues on cadmium-induced mice [51]. In this study, EFEL decreased the activity of AChE and increased the content of ACh in PM_2.5_-induced cholinergic dysfunction. In addition, EFEL regulated the expression level of AChE and ChAT in the whole brain and hippocampus tissues. Therefore, it was confirmed that EFEL improves cognitive impairment by regulating the cholinergic system in PM_2.5_-induced neurotoxicity.

PM_2.5_ can damage the mitochondrial structure and ATP synthesis metabolite [52]. In a previous study, exposure to PM_2.5_ increased the expression of genes that induce mitochondrial fission in the nasal mucosa of rat [53]. In this study, the intake of EFEL ameliorated excessive ROS production and loss of MMP and ATP in the lung and brain mitochondria of PM_2.5_-induced mice. Exposure to PM_2.5_ decreased membrane potential and increased intracellular ROS levels in human bronchial epithelial cells (BEAS-2B) [54]. In addition, the bark extract of *Eucommia ulmoides* reduced the ROS level and improved the loss of MMP in H_2_O_2_-induced human neuroblastoma cells (SH-SY5Y) [55]. The rutin contained in *Eucommia ulmoides* improved the sodium nitroprusside-induced reduction of MMP in PC12 cells [56]. In addition, the quercetin contained in *Eucommia ulmoides* increased the SOD and ATP contents and improved the loss of MMP in the mitochondria of chloral hydrate-induced traumatic brain injury mice [57]. As a result, PM_2.5_ exposure induced mitochondrial dysfunction of lung and brain tissue, and EFEL reduced ROS production and inhibited the loss of MMP and ATP levels in mitochondria.

PM_2.5_ entering through the respiratory tract induces oxidative stress and inflammatory responses, which can secrete various inflammatory cytokines such as interleukin (IL)-1, IL-16, MCP-1, TNF-*α*, and C(cysteine)-X(noncysteine)-C(cysteine) motif (CXC) chemokines such as CXC chemokine ligand 1(CXCL1) and CXC chemokine ligand 8 (CXCL8) in lung tissue [58, 59]. PM_2.5_ and inflammatory cytokines circulating in the whole body pass the BBB and attack brain tissue, and PM_2.5_ deposited in the olfactory bulb promotes neuroinflammation [60, 61]. The excessive inflammatory response in the brain disrupts the neurotransmitter system, which leads to hippocampus damage to learning and memory [62]. The stimulation of TLR4 as a membrane receptor increases the expression level of phosphorylation extracellular signal-related kinase (ERK), c-Jun N-terminal kinase (JNK), and *p*-NF*κ*B and *p*-I*κ*B*α*, stimulating the secretion of inflammatory cytokines such as IL-1*β* and TNF-*α* [63]. Also, PM_2.5_ exposure increases the activity of poly (ADP-ribose) polymerase (PARP)-1, which promotes the formation of amyloid-*β*, and shows excessive activity of glial cells as evidence of neuroinflammation [64]. Exposure to PM_2.5_ increased the levels of soluble and insoluble amyloid-*β* and phosphorylated tau protein in mouse brain tissue [65, 66]. In this study, EFEL reduced the expression levels of protein related to inflammatory responses in the lung and brain tissues of mice and decreased the expression levels of amyloid-*β* and *p*-tau in the brain tissue. Polysaccharides extracted from *Eucommia ulmoides* leaves regulated the TLR4-NF*κ*B pathway in hepatic ischemia-reperfusion injury mice [67]. *Eucommia ulmoides* bark reduced the expression level of *p*-JNK, *p*-ERK, TNF-*α*, and IL-1*β* in LPS-induced microglial BV-2 cells [68]. Also, rutin as a physiological compound of EFEL decreased the expression of TNF-*α*, IL-1*β*, IL-6, *p*-ERK, and *p*-JNK and decreased the expression level of I*κ*B in LPS-induced lung injury mice [69]. Quercetin contained in *Eucommia ulmoides* decreased the expression levels of *p*-ERK, *p*-JNK, and *p*-p65 in okadaic acid-induced hippocampal neurons and reduced the expression levels of amyloid-*β*_1-42_ and phosphorylation tau protein in aged triple transgenic Alzheimer's disease model mice [70, 71]. In this study, EFEL down-regulated the expression levels of *p*-JNK, *p*-I*κ*B*α*, caspase-1, IL-1*β*, and TNF-*α* in the lung and the expression levels of *p*-JNK, *p*-I*κ*B*α*, caspase-1, IL-1*β*, and TNF-*α* in olfactory bulb caused by PM_2.5_ toxicity. These results suggest that the regulation of pulmonary inflammation decreases the inflammatory factor passing through the blood, thereby reducing the expression of the neuroinflammatory factor in hippocampus tissues. In addition, EFEL reduced the expression levels of amyloid-*β* and *p*-tau in the brain tissue. Therefore, EFEL can be used as a functional food material to improve PM_2.5_-induced inflammatory response and cognitive impairment by regulating inflammation.

## 5. Conclusions

In this study, the protective effect of the ethyl acetate fraction of *Eucommia ulmoides* leaves (EFEL) against PM_2.5_-induced excessive inflammation and cognitive impairment in BALB/c mice was confirmed. EFEL increased the level of spontaneous alternation behavior and protected the long-term memory ability of PM_2.5_-induced mice. EFEL inhibited the antioxidant deficit and mitochondrial damage in PM_2.5_-induced lung and brain tissues and attenuated cerebral cholinergic dysfunction. EFEL regulated the protein expression level related to inflammation in the lung and brain tissues. Inhibition of inflammation in lung tissue by EFEL indicates reduced neuroinflammation in the whole brain, olfactory bulb, and hippocampus, which improved the learning and memory decline in mice. EFEL inhibited damage to mitochondria and antioxidant systems in lung and brain tissues, and consequently protected against the cognitive impairment induced by PM_2.5_. In conclusion, it is suggested that *Eucommia ulmoides* leaves could be used as a material for functional food to improve PM_2.5_-induced cognitive impairment by regulating the inflammatory response.

## Figures and Tables

**Table 1:** Bioactive compounds identified from ethyl acetate fraction of *Eucommia ulmoides* leaves (EFEL).

No.	RT (min)	Parent Ion (m/z)	MS^2^ fragment (m/z)	Compound
1	2.84	707	354, 353	5-O-Caffeoylquinic acid
2	3.15	609	301	Rutin
3	3.22	463	301	Quercetin-O-hexoside
4	3.28	505	301, 300	Quercetin-O-acetyl hexoside (isomer)
5	3.44	489	285	Luteolin-O-acetyl hexoside
6	3.84	301	179, 151	Quercetin

## Data Availability

The research data used to support the findings of this study are included within the article.

## Supplementary Materials

Supplementary Materials Figure 1: EFEL increased the cell viability on PM_2.5_-induced cytotoxicity of RPMI 2650 (nasal cell), A549 (lung cell), HT22 (hippocampus cell), MC-IXC (human neuronal cells), and BV-2 (microglia cell) cells. Figure 2 EFEL inhibited the ROS formation on PM_2.5_-induced cytotoxicity of RPMI 2650 (nasal cell), A549 (lung cell), HT22 (hippocampus cell), MC-IXC (human neuronal cells), and BV-2 (microglia cell) cells.
