# Supplementary material for: Effect of Ethyl Acetate Fraction from Eucommia ulmoides Leaves on PM2.5-Induced Inflammation and Cognitive Dysfunction
Source: Oxid Med Cell Longev. 2022 May 14;2022:7157444. doi: 10.1155/2022/7157444 (PMC9124148; doi:10.1155/2022/7157444)
Supplement: Supplementary Materials — Figure 1: EFEL increased the cell viability on PM2.5-induced cytotoxicity of RPMI 2650 (nasal cell), A549 (lung cell), HT22 (hippocampus cell), MC-IXC (human neuronal cells), and BV-2 (microglia cell) cells. Figure 2 EFEL inhibited the ROS formation on PM2.5-induced cytotoxicity of RPMI 2650 (nasal cell), A549 (lung cell), HT22 (hippocampus cell), MC-IXC (human neuronal cells), and BV-2 (microglia cell) cells. [file 7157444.f1.docx]

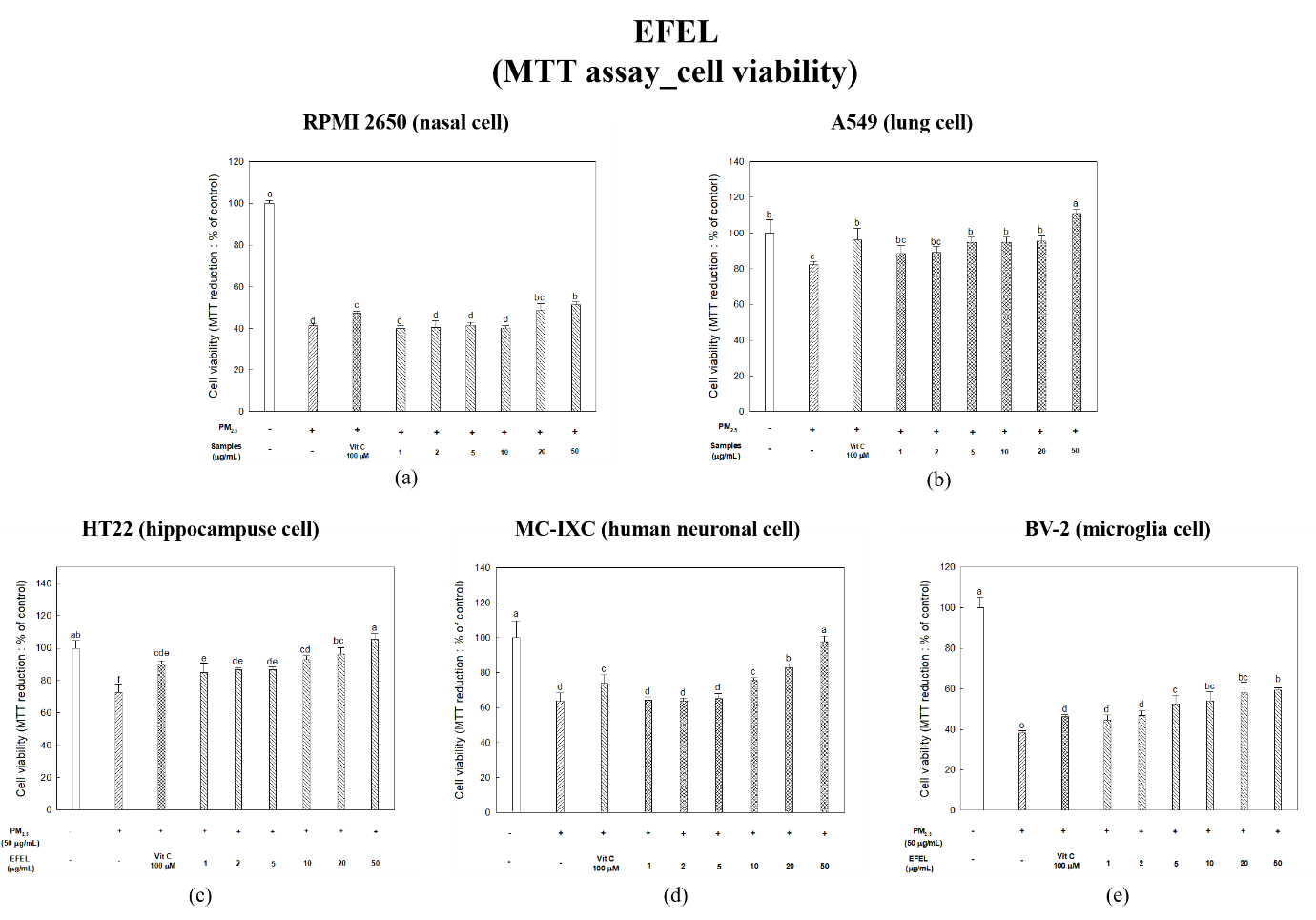


Supplemental Figure 1. Cell protective effect of EFEL on PM_2.5_-induced (a) RPMI2650; (b) A549; (c) HT22; (d) MC-IXC; (e) BV-2 cells. Results shown are mean ± SD (n=3). Data were statistically represented at *p*<0.05, and different lowercase letters indicate statistical significance.


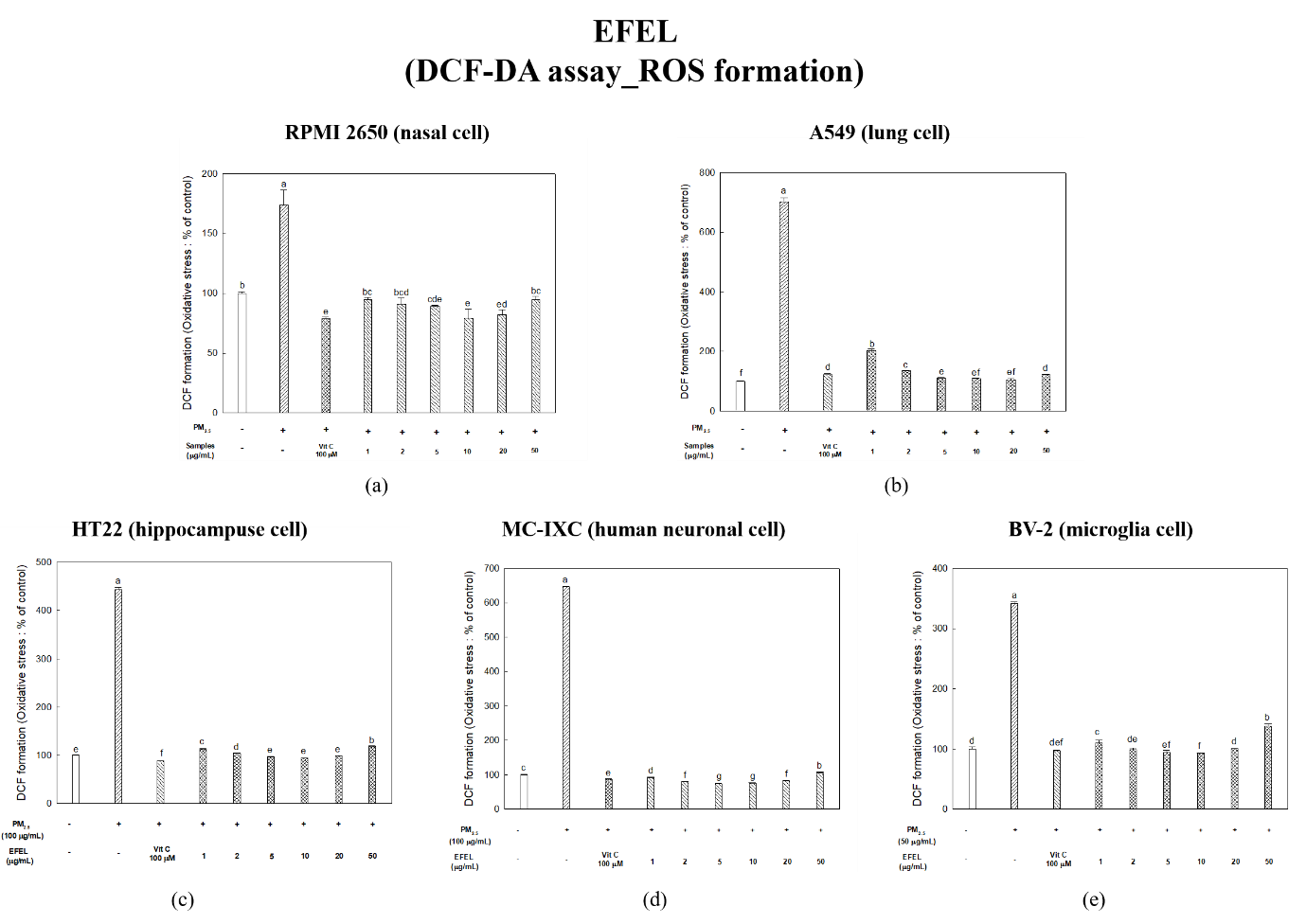


Supplemental Figure 2. Reactive oxygen species (ROS) inhibition effect of EFEL on PM_2.5_-induced (a) RPMI2650; (b) A549; (c) HT22; (d) MC-IXC; (e) BV-2 cells. Results shown are mean ± SD (n=3). Data were statistically represented at *p*<0.05, and different lowercase letters indicate statistical significance.
